# Supplementary material for: HBM4EU Chromates Study: Determinants of Exposure to Hexavalent Chromium in Plating, Welding and Other Occupational Settings
Source: Int J Environ Res Public Health. 2022 Mar 19;19(6):3683. doi: 10.3390/ijerph19063683 (PMC8953290; doi:10.3390/ijerph19063683)
Supplement: Supplementary file 1 [file ijerph-19-03683-s001.zip › ijerph-1615924-supplementary.pdf]

Supplementary Material of the manuscript: HBM4EU Chromates study: Determinants of exposure to hexa-valent chromium in plating, welding and other occupational settings

Table S1. Main characteristics of the companies enrolled in the study.

Questionnaire		Number of workers	%
**Companies**	**Company classification by industrial sector**		
	Metallurgy	160	40.1
	Steel and steel products/Metals	60	15.0
	Food Industry	39	9.8
	Electroplating	33	8.3
	Mechanics	34	8.5
	Heavy industry	17	4.3
	Engineering Industry	15	3.8
	Aircraft maintenance	13	3.3
	Chrome plating	13	3.3
	Shipbuilding industry	11	2.8
	Electronics	4	1.0
	**Previous monitoring actions**		
	Environment and biomonitoring	283	70.9
	Environment (air and/or dermal samples)	33	8.3
	Biomonitoring	0	0.0
	None	68	17.0
	Do not know	15	3.8
**Workers**	**Work activity**		
	Welding	195	48.9
	Bath plating	90	22.6
	Painting	52	13.0
	Machining	38	9.5
	Steel production	11	2.8
	Maintenance and laboratory work	8	2.0

Thermal spraying	5	1.3
**Site**		
Indoor work	383	96.0
Outdoor work	7	1.8
No predominant site	8	2.0
**Duration of work shifts**		
< 7 hours	0	0.0
7 - 8 hours	43	10.8
8 hours	315	78.9
> 8 hours	40	10.0
**Type of work shifts**		
Fixed day	243	60.9
Other	53	13.3
Rotating day/back/night	50	12.5
Rotating day/back	33	8.3
Fixed night	20	5.0
**Previous training in OSH issues**		
Yes	334	83.7
No	25	6.3
Missing data	40	10.0
**Possibility of hands washing during work shift**		
Yes	389	97.5
Missing data	10	2.5
**Dedicated place for storing working clothes**		
Yes	332	83.2
No	51	12.8
Missing data	16	4.0
**Dedicated place for storing RPE**		
Yes	251	62.9
No	53	13.3
Missing data	95	23.8

Table S2. Characterization of workers.

	All Workers (n=399)		Welding (n=195)		Bath Plating (n=90)		Machining (n=38)		Painting (n=52)		Steel production (n=11)		Thermal spraying (n=5)		Maintenance and Laboratory work (n=8)	
	**n**	**%**	**n**	**%**	**n**	**%**	**n**	**%**	**n**	**%**	**n**	**%**	**n**	**%**	**n**	**%**
**Sex**																
Male	390	97.7	194	99.5	88	97.8	38	100.0	49	94.2	11	100.0	5	100.0	5	62.5
Female	9	2.2	1	0.5	2	2.2	0	0.0	3	5.8	0	0.0	0	0.0	3	37.5
**Country**																
Belgium	71	17.8	28	14.4	8	8.9	9	23.7	15	28.8	11	100.0	0	0.0	0	0.0
Finland	50	12.5	25	12.8	18	20.0	2	5.3	0	0.0	0	0.0	5	100.0	0	0.0
France	58	14.5	18	9.2	20	22.2	19	50.0	0	0.0	0	0.0	0	0.0	1	12.5
Italy	48	12.0	38	19.5	6	6.7	0	0.0	4	7.7	0	0.0	0	0.0	0	0.0
Luxembourg	16	4.0	16	8.2	0	0.0	0	0.0	0	0.0	0	0.0	0	0.0	0	0.0
The Netherlands	20	5.0	0	0.0	19	21.1	0	0.0	0	0.0	0	0.0	0	0.0	1	12.5
Poland	52	13.0	51	26.2	0	0.0	0	0.0	1	1.9	0	0.0	0	0.0	0	0.0
Portugal	50	12.5	3	1.5	5	5.6	5	13.2	32	61.5	0	0.0	0	0.0	5	62.5
United Kingdom	34	8.5	16	8.2	14	15.6	3	7.9	0	0.0	0	0.0	0	0.0	1	12.5
**Age**																
20-29 years	58	14.5	38	19.5	9	10.0	6	15.8	2	3.8	2	18.2	2	40.0	1	12.5
30-39 years	117	29.3	57	29.2	25	27.8	10	26.3	22	42.3	0	0.0	0	0.0	1	12.5
40-49 years	102	25.6	41	21.0	27	30.0	11	28.9	15	28.8	6	54.5	1	20.0	1	12.5
50-59 years	90	22.6	47	24.1	18	20.0	10	26.3	8	15.4	3	27.3	1	20.0	3	37.5
60-68 years	19	4.8	6	3.1	8	8.9	1	2.6	1	1.9	0	0.0	1	20.0	2	25.0
Missing data	13	3.3	6	3.1	3	3.3	0	0.0	4	7.7	0	0.0	0	0.0	0	0.0
**Smoking status**																
Smoker	140	35.6	64	33.0	40	44.4	13	34.2	16	30.8	3	27.3	2	40.0	2	25.0
Non-smoker	155	39.4	73	37.4	32	35.6	13	34.2	27	51.9	6	54.5	1	2.0	3	37.5
Former smoker	98	24.6	57	29.2	17	18.9	12	31.6	5	9.6	2	18.2	2	40.0	3	37.5
Missing data	6	1.5	1	0.5	1	1.1	0	0	4	7.7	0	0.0	0	0.0	0	0.0

**Home location**																
Urban	254	63.7	123	63.1	66	73.4	18	47.4	36	69.2	1	9.1	4	80.0	6	75.0
Rural	135	33.8	70	35.9	21	23.3	19	50.0	12	23.1	10	90.9	1	20.0	2	25.0
Missing data	10	2.5	2	1.0	3	3.3	1	2.6	4	7.7	0	0.0	0	0.0	0	0.0
**Home - traffic density**																
Low density	208	52.1	108	55.4	55	61.1	18	47.4	15	28.8	9	81.8	2	40.0	1	12.5
Medium density	133	33.3	63	32.3	22	24.4	15	39.5	23	44.2	1	9.1	2	40.0	7	87.5
Heavy density	52	13.0	23	11.8	12	13.3	5	13.2	10	19.2	1	9.1	1	20.0	0	0.0
Missing data	6	1.6	1	0.5	1	1.1	0	0.0	4	7.7	0	0.0	0	0.0	0	0.0
	**Mean ± SD**	**Max**	**Mean ± SD**	**Max**	**Mean ± SD**	**Max**	**Mean ± SD**	**Max**	**Mean ± SD**	**Max**	**Mean ± SD**	**Max**	**Mean ± SD**	**Max**	**Mean ± SD**	**Max**
**Years of experience**																
Metal plating	15.4 ± 11.7	46	11.0 ± 14.1	21	15.9 ± 11.5	46	8.3 ± 5.5	13	12.5 ± 0.7	13	NR	NR	5.5 ± 2.6	8	23.0 ± 15.4	41
Painting or spraying	11.9 ± 9.6	42	2.0 *	NR	1.5 ± 0.7	2	13.7 ± 4.9	21	11.8 ± 11.5	42	NR	NR	NR	NR	NR	NR
Welding	14.7 ± 12.2	47	15.2 ± 12.4	47	7.6 ± 5.2	15	6.5 ± 6.4	11	10.0 *	10	NR	NR	NR	NR	NR	NR
Other metal works	14.2 ± 10.0	39	9.5 ± 7.8	25	9.6 ± 6.6	22	16.8 ± 12.6	39	19.9 ± 8.0	33	24.3 ± 5.6	33	22.0 *	22.0	25.0 *	25.0

Table S3. Detailed characteristics regarding the use of RPE, gloves, coveralls and other protective equipment, and presence of local exhaustion ventilation (LEV), for welding.

Activity - Welding	Manual welding		Manual tack-welding		Robot welding		Other manual tasks: cleaning, grinding, cutting		Cleaning and maintenance of equipment		Waste management		Other tasks	
	n= 149		n=38		n=28		n=127		n=42		n=29		n=43	
	n	%	n	%	n	%	n	%	n	%	n	%	n	%
**RPE**														
Welding helmet with half mask re-usable dust respirator	22	14.8	1	2.6	1	3.6	17	13.4	0	0.0	1	3.4	0	0.0
Welding helmet with disposable particulate respirator	14	9.4	5	13.2	1	3.6	0	0.0	0	0.0	2	6.9	0	0.0
Welding helmet with powered or air-fed filtering respirator	51	34.2	5	13.2	3	10.7	25	19.7	0	0.0	0	0.0	1	2.4
Welding helmet without any respirator	57	38.3	18	47.4	9	32.1	7	5.5	0	0.0	0	0.0	2	4.8
Welding helmet with other respiratory protection equipment	0	0.0	1	2.6	5	17.9	1	0.8	0	0.0	0	0.0	0	0.0
Powered or air-fed filtering respirator	0	0.0	0	0.0	0	0.0	10	7.9	1	2.4	1	3.4	0	0.0
Reusable half or full-face mask respirator (without powered or air-fed respirator)	0	0.0	0	0.0	0	0.0	8	6.3	0	0.0	0	0.0	0	0.0
Other RPE	0	0.0	1	2.6	2	7.1	15	11.8	7	16.7	0	0.0	4	9.5
No RPE	1	0.7	3	7.9	1	3.6	13	10.2	6	14.3	2	6.9	15	35.7
No available data	4	2.7	4	10.5	6	21.4	31	24.4	28	66.7	23	79.3	21	50.0
**Gloves**														
Welding gloves	115	77.2	21	55.3	14	50.0	63	49.6	9	21.4	9	31.0	3	7.1
Welding gloves and other gloves	4	2.7	0	0.0	8	28.6	4	3.1	0	0.0	0	0.0	2	4.8
Other gloves	3	2.0	2	5.3	0	0	27	21.3	1	2.4	1	3.4	26	61.9
No gloves	4	2.7	3	7.9	0	0	5	3.9	1	2.4	1	3.4	0	0.0
No available data	23	15.4	12	31.6	6	21.4	28	22.0	31	73.8	18	62.1	17	40.5
**Coveralls and other protective equipment**														
Fire/flame resistant clothing	74	49.7	8	21.1	13	46.4	52	40.9	6	14.3	6	20.7	4	9.5
Fire/flame resistant clothing, hearing protection	5	3.4	0	0.0	0	0.0	0	0.0	0	0.0	0	0.0	0	0.0
Fire/flame resistant clothing, safety footwear	5	3.4	1	2.6	0	0.0	5	3.9	0	0.0	0	0.0	0	0.0
Fire/flame resistant clothing, protective glasses	0	0.0	0	0.0	0	0.0	4	3.1	0	0.0	0	0.0	0	0.0
Fire/flame resistant clothing, leather apron	0	0.0	0	0.0	0	0.0	1	0.8	0	0.0	0	0.0	0	0.0
Fire/flame resistant clothing, visor	0	0.0	0	0.0	0	0.0	1	0.8	0	0.0	0	0.0	0	0.0
Protective glasses	0	0.0	1	2.6	0	0.0	5	3.9	0	0.0	0	0.0	2	4.8
Protective glasses, apron	0	0.0	1	2.6	0	0.0	0	0.0	0	0.0	0	0.0	0	0.0
Protective glasses, hearing protection	0	0.0	0	0.0	0	0.0	2	1.6	0	0.0	0	0.0	0	0.0

Coveralls	0	0.0	0	0.0	3	10.7	0	0.0	0	0.0	0	0.0	1	2.4
Other workwear	8	5.4	0	0.0	1	3.6	0	0.0	0	0.0	0	0.0	4	9.5
No available data	57	38.3	17	44.7	11	39.3	57	44.9	36	85.7	23	79.3	32	76.2
**Ventilation - LEV**														
Extracted welding booth	42	28.2	1	2.6	3	10.7	0	0.0	0	0.0	0	0.0	0	0.0
Extracted work bench	0	0.0	3	7.9	0	0.0	1	0.8	0	0.0	0	0.0	0	0.0
Movable welding hood	0	0.0	2	5.3	3	10.7	10	7.9	0	0.0	0	0.0	1	2.4
Extracted work bench, general ventilation	0	0.0	0	0.0	1	3.6	1	0.8	0	0.0	0	0.0	0	0.0
Gun fixed extraction, general ventilation	0	0.0	0	0.0	2	7.1	0	0.0	0	0.0	0	0.0	0	0.0
Movable welding hood, extracted work bench, general ventilation	0	0.0	0	0.0	0	0.0	5	3.9	0	0.0	0	0.0	0	0.0
General ventilation	0	0.0	9	23.7	10	35.7	52	40.9	10	23.8	10	34.5	14	33.3
Other LEV	63	42.3	2	5.3	0	0.0	2	1.6	0	0.0	0	0.0	0	0.0
No available data	44	29.5	21	55.3	9	32.1	57	44.9	32	76.2	19	65.5	28	66.7

RPE = Respiratory protective equipment; LEV = Local exhaustion ventilation; n= number of workers performing the task.

Table S4. Detailed characteristics regarding the use of RPE, gloves, coveralls and other protective equipment, and presence of local exhaustion ventilation (LEV), for bath plating.

Activity – Bath plating	Readjustment		Chromate Electroplating dipping		Occasional maintenance activities		Bath sampling		Laboratory analysis		Waste management		Other tasks	
	n= 33		n=84		n=25		n=7		n=5		n=11		n=23	
	n	%	n	%	n	%	n	%	n	%	n	%	n	%
**Process type**														
Manual	24	72.7	67	79.8	22	88.0	7	100.0	4	80.0	9	81.8	14	60.9
Automatic	8	24.2	16	19.0	0	0.0	0	0.0	0	0.0	1	9.1	1	4.3
No available data	1	3.0	1	1.2	3	12.0	0	0.0	1	20.0	1	9.1	8	34.8
**RPE**														
Disposable face mask	2	6.1	3	3.6	1	4.0	0	0.0	0	0.0	0	0.0	1	4.3
Powered or air-fed. filtering respirator	4	12.1	4	4.8	2	8.0	3	42.9	1	20.0	1	9.1	2	8.7
Reusable half or full-face mask respirator (without powered or air-fed respirator)	4	12.1	6	7.1	3	12.0	0	0.0	0	0.0	0	0.0	0	0.0
No RPE	18	54.5	57	67.9	11	44.0	4	57.1	3	60.0	3	27.3	10	43.5
No available data	5	15.2	14	16.7	8	32.0	0	0.0	1	20.0	7	63.6	10	43.5
**Gloves**														
Disposable gloves	13	39.4	30	35.7	12	48.0	3	42.9	4	80.0	7	63.6	8	34.8
Reusable gloves	10	30.3	30	35.7	3	12.0	2	28.6	0	0.0	0	0.0	5	21.7
Reusable and disposable gloves	7	21.2	17	20.2	4	16.0	1	14.3	0	0.0	0	0.0	1	4.3
No gloves	0	0.0	1	1.2	0	0.0	1	14.3	0	0.0	1	9.1	2	8.7
No available data	3	9.1	6	7.1	6	24.0	0	0.0	1	20.0	3	27.3	7	30.4
**Coveralls and other protective equipment**														
Protective glasses	5	15.1	22	25.9	2	8.0	0	0.0	1	20.0	1	9.1	2	8.7
Protective glasses, hearing protection	0	0.0	1	1.2	2	8.0	0	0.0	0	0.0	0	0.0	1	4.3
Protective glasses, coveralls	3	9.1	6	7.1	1	4.0	2	28.6	2	40.0	0	0.0	3	7.9
Protective glasses, visor	0	0.0	2	2.4	0	0.0	0	0.0	0	0.0	0	0.0	0	0.0
Protective glasses, helmet	1	3.0	0	0.0	0	0.0	0	0.0	0	0.0	0	0.0	0	0.0
Protective glasses, helmet, coveralls	3	9.1	4	4.7	4	16.0	2	28.6	1	20.0	2	18.2	1	4.3
Protective glasses, protection suit, safety footwear	9	27.3	11	13.1	2	8.0	0	0.0	0	0.0	1	7.7	3	13.0
Protective glasses, coveralls, safety footwear	0	0.0	5	5.9	0	0.0	0	0.0	0	0.0	0	0.0	0	0.0
Protective glasses, coveralls, safety footwear, hearing protection	0	0.0	1	1.2	0	0.0	0	0.0	0	0.0	0	0.0	0	0.0

Protective glasses, visor, helmet, long sleeves, hearing protection, safety footwear	0	0.0	1	1.2	0	0.0	0	0.0	0	0.0	0	0.0	0	0.0
Coveralls	0	0.0	1	1.2	1	4.0	0	0.0	0	0.0	0	0.0	1	4.3
Coveralls, disposable gloves, glasses, safety shoes	0	0.0	1	1.2	0	0.0	0	0.0	0	0.0	0	0.0	0	0.0
Coveralls, helmet	0	0.0	2	2.4	0	0.0	0	0.0	0	0.0	1	9.1	1	4.3
Protection suit	0	0.0	2	2.4	0	0.0	0	0.0	0	0.0	0	0.0	0	0.0
Visor	0	0.0	3	3.5	0	0.0	0	0.0	0	0.0	0	0.0	0	0.0
Acid resistant protection suit	0	0.0	6	7.1	6	24.0	0	0.0	0	0.0	0	0.0	0	0.0
No available data	12	36.4	16	18.8	7	28.0	3	42.9	7	20.0	7	63.7	11	47.8
**Ventilation - LEV**														
Yes	17	51.5	54	64.3	13	52.0	3	42.9	3	60.0	3	27.3	8	34.8
No	9	27.3	18	21.4	6	24.0	2	28.6	1	20.0	5	45.5	8	34.8
No available data	7	21.2	12	14.3	6	24.0	2	28.6	1	20.0	3	27.3	7	30.4

RPE = Respiratory protective equipment; LEV = Local exhaustion ventilation; n= number of workers performing the task.

Table S5. Detailed characteristics regarding the use of RPE, gloves, coveralls and other protective equipment, and presence of local exhaustion ventilation (LEV), for painting.

Activity - Painting	Preparation tasks: decanting. mixing of paints. re-filling of apparatus		Spraying in spray cabin/spray booth		Spraying outside of spray booth		Surface treatment in automatic spray tunnel		Surface treatment by brushing or pen stick		Drying/self-curing with no LEV		Cleaning and maintenance of equipment		Occasional maintenance activities		Machining operations (grinding) on parts containing chromium	Waste management	Other tasks	
	n= 24		n=23		n=10		n=5		n=14		n=6		n=34		n=8		n=1	n=1	n=28	
**RPE**																				
Disposable face mask	0	0.0	1	4.3	0	0.0	0	0.0	1	7.2	1	16.7	0	0.0	0	0.0	0	0	4	14.3
Powered or air-fed. filtering respirator	1	4.2	0	0.0	0	0.0	0	0.0	1	7.2	0	0.0	8	23.6	0	0.0	1	0	0	0.0
Reusable half or full-face mask respirator (without powered or air-fed respirator)	22	91.6	22	95.7	9	90.0	2	40.0	10	71.4	0	0.0	24	70.6	0	0.0	0	0	15	53.6
No RPE	0	0.0	0	0.0	0	0.0	0	0.0	2	14.2	4	66.6	1	2.9	1	12.5	0	1	8	28.6
No available data	1	4.2	0	0.0	1	10.0	3	60.0	0	0.0	1	16.7	1	2.9	7	87.5	0	0	1	3.6
**Gloves**																				
Disposable gloves	12	50.0	11	47.8	5	50.0	1	20.0	6	42.8	0	0.0	12	35.3	0	0.0	0	0	14	50.0
Reusable gloves	9	37.4	10	43.6	5	50.0	2	40.0	7	50.0	0	0.0	19	55.9	8	100.0	1	1	12	42.8
Reusable and disposable gloves	1	4.2	1	4.3	0	0.0	0	0.0	0	0.0	0	0.0	1	2.9	0	0.0	0	0	0	0.0
No gloves	1	4.2	0	0.0	0	0.0	0	0.0	1	7.2	4	66.6	0	0.0	0	0.0	0	0	1	3.6
No available data	1	4.2	1	4.3	0	0.0	2	40.0	0	0.0	2	34.4	2	5.8	0	0.0	0	0	1	3.6
**Coveralls and other protective equipment**																				
Protective glasses	8	33.3	2	8.7	1	10.0	1	20.0	3	21.6	0	0.0	12	35.3	8	100.0	0	0	0	0.0
Protective glasses. coveralls	0	0.0	0	0.0	0	0.0	0	0.0	0	0.0	0	0.0	0	0.0	0	0.0	0	0	1	3.6
Protective glasses. protection suit	2	8.4	7	30.4	4	40.0	1	20.0	1	7.2	0	0.0	2	5.8	0	0.0	0	0	2	7.2
Coveralls	0	0.0	0	0.0	0	0.0	0	0.0	0	0.0	0	0.0	0	0.0	0	0.0	0	0	7	25.0
Protection suit	5	20.9	9	39.1	4	40.0	0	0.0	4	28.8	0	0.0	8	23.6	0	0.0	0	0	12	42.8
Hearing protection	0	0.0	0	0.0	0	0.0	0	0.0	0	0.0	0	0.0	0	0.0	0	0.0	0	0	1	3.6

No available data	9	37.4	5	21.7	1	10.0	3	30.0	6	43.2	6	100.0	12	35.3	0	0.0	1		1		5	17.9
**Ventilation - LEV**																						
Yes	15	62.6	21	91.4	0	0.0	2	40.0	2	14.3	0	0.0	22	64.7	0	0.0	0		0		12	42.8
No	7	29.2	1	4.3	10	100.0	0	0.0	11	78.5	5	83.3	10	29.4	8	100.0	1		1		14	50.0
No available data	2	8.2	1	4.3	0	0.0	3	60.0	1	7.2	1	16.7	2	2.9	0	0.0	0		0		2	7.2

RPE = Respiratory protective equipment; LEV = Local exhaustion ventilation; n= number of workers performing the task.

Table S6. Detailed characteristics regarding the use of RPE, gloves, coveralls and other protective equipment, and presence of local exhaustion ventilation (LEV), for machining.

Activity - Machining	Preparation tasks: decanting. mixing of paints. re-filling of apparatus		Spraying in spray cabin/spray booth		Surface treatment by rolling		Surface treatment by brushing or pen stick		Drying/self-curing with no LEV		Cleaning and maintenance of equipment		Occasional maintenance activities		Machining operations (grinding) on parts containing chromium		Machining operations (grinding) on parts covered with chromium paint		Waste management		Other tasks		Readjustment	Application in baths	Other tasks	
	n= 9		n=4		n=12		n=14		n=8		n=12		n=4		n=13		n=10		n=15		n=10		n=1	n=1	n=14	
**RPE**																										
Disposable face mask	0	0.0	0	0.0	1	8.3	1	7.1	1	12.5	0	0.0	1	25.0	3	23.1	1	10.0	0	0.0	0	0.0	0	0	0	0.0
Powered or air-fed. filtering respirator	3	33.3	2	50.0	0	0.0	1	7.1	0	0.0	1	8.3	1	25.0	0	0.0	0	0.0	0	0.0	0	0.0	0	0	0	0.0
Reusable half or full-face mask respirator (without powered or air-fed respirator)	1	11.1	1	25.0	4	33.5	7	50.0	3	37.5	0	0.0	0	0.0	5	38.5	5	50.0	2	13.3	5	50.0	0	1	3	21.4
Reusable half or full-face mask respirator	0	0.0	0	0	0	0.0	0	0.0	0	0.0	0	0.0	0	0.0	0	0.0	0	0.0	0	0.0	0	0.0	1	0	0	0.0
No RPE	0	0.0	0	0.0	0	0.0	0	0.0	0	0.0	0	0.0	0	0.0	1	7.7	0	0.0	0	0.0	0	0.0	0	0	1	7.1
No available data	5	55.6	1	25.0	7	58.3	5	35.7	4	50.0	11	91.7	2	50.0	4	30.7	4	40.0	13	86.7	5	50.0	0	0	10	71.5
**Gloves**																										
Disposable gloves	5	55.6	2	50.0	5	41.7	6	42.9	4	50.0	3	25.0	1	25.0	5	38.5	5	50.0	6	39.9	6	60.0	0	0	5	35.7
Reusable gloves	2	22.2	2	50.0	2	16.6	1	7.1	1	12.5	0	0.0	1	25.0	2	15.4	1	10.0	0	0.0	1	10.0	1	1	0	0.0
Reusable and disposable gloves	0	0.0	0	0.0	1	8.3	0	0.0	0	0.0	0	0.0	0	0.0	0	0.0	0	0.0	0	0.0	1	10.0	0	0	0	0.0
Cut resistant gloves	0	0.0	0	0.0	0	0.0	0	0.0	0	0.0	0	0.0	0	0.0	0	0.0	0	0.0	1	6.6	0	0.0	0	0	0	0.0
No gloves	0	0.0	0	0.0	0	0.0	1	7.1	0	0.0	0	0.0	0	0.0	2	15.4	0	0.0	0	0.0	0	0.0	0	0	9	64.3
No available data	2	22.2	0	0.0	4	33.5	6	42.9	3	37.5	9	75.0	2	50.0	4	30.8	4	40.0	8	61.5	2	20.0	0	0	0	0.0
**Coveralls and other protective equipment**																										
Protective glasses	0	0.0	0	0.0	0	0.0	0	0.0	0	0.0	0	0.0	1	25.0	4	30.8	0	0.0	0	0.0	0	0.0	0	0	0	0.0

Protective glasses, protection suit, safety footwear	0	0.0	0	0.0	0	0.0	0	0.0	0	0.0	0	0.0	0	0.0	0	0.0	0	0.0	0	0.0	0	0.0	1	1	3	21.4
Protective glasses, protection suit, safety footwear, hearing protection	0	0.0	0	0.0	0	0.0	0	0.0	0	0.0	0	0.0	0	0.0	2	15.4	0	0.0	0	0.0	0	0.0	0	0	0	0.0
Coveralls	0	0.0	1	25.0	1	8.3	0	0.0	0	0.0	0	0.0	0	0.0	0	0.0	0	0.0	0	0.0	0	0.0	0	0	0	0.0
Visor	0	0.0	0	0.0	0	0.0	0	0.0	0	0.0	0	0.0	0	0.0	0	0.0	0	0.0	0	0.0	1	10.0	0	0	0	0.0
No available data	9	100.0	3	75.0	11	91.7	14	100.0	8	100.0	12	100.0	3	75.0	7	53.8	10	100.0	15	100.0	9	90.0	0	0	11	78.6
**Ventilation - LEV**																										
Yes	4	44.4	4	100.0	3	25.0	1	7.1	2	25.0	2	16.6	2	50.0	1	7.7	1	10.0	2	13.3	2	20.0	0	1	4	28.6
No	5	55.6	0	0.0	7	66.7	12	85.8	5	62.5	7	58.4	1	25.0	11	84.6	9	90.0	9	60.1	6	60.0	1	0	9	64.3
No available data	0	0.0	0	0.0	1	8.3	1	7.1	1	12.5	3	25.0	1	25.0	1	7.7	0	0.0	4	26.6	2	20.0	0	0	1	7.1

RPE = Respiratory protective equipment; LEV = Local exhaustion ventilation; n= number of workers performing the task.

Table S7. Detailed characteristics regarding the use of RPE, gloves, coveralls and other protective equipment, and presence of local exhaustion ventilation (LEV), for steel production.

Activity – Steel production	Cleaning and maintenance of equipment	Occasional maintenance activities	Other tasks
	**n=2**	**n=5**	**n=8**
**RPE**			
Disposable face mask	0	0	0
Powered or air-fed. filtering respirator	0	0	0
Reusable half or full-face mask respirator (without powered or air-fed respirator)	0	0	8
Reusable half or full-face mask respirator	0	0	0
No RPE	2	5	0
No available data	0	0	0
**Gloves**			
Disposable gloves	0	0	0
Reusable gloves	2	5	8
Reusable and disposable gloves	0	0	0
Cut resistant gloves	0	0	0
No gloves	0	0	0
No available data	0	0	0
**Coveralls and other protective equipment**			
Protective glasses	0	0	0
Protective glasses, protection suit, safety footwear	0	0	0
Protective glasses, coveralls, safety footwear, hearing protection	0	0	0
Coveralls	2	5	8
Visor	0	0	0
Acid resistant protection suit	0	0	0
No available data	0	0	0
**Ventilation - LEV**			
Yes	0	0	0
No	2	5	8
No available data	0	0	0

RPE = Respiratory protective equipment; LEV = Local exhaustion ventilation; n= number of workers performing the task.

Table S8. Detailed characteristics regarding the use of RPE, gloves, coveralls and other protective equipment, and presence of local exhaustion ventilation (LEV), for maintenance and laboratory work

Activity – Maintenance and laboratory work	Readjustment	Infrequent maintenance activities	Sampling	Laboratory analysis	Waste management	Other tasks	Occasional maintenance activities
	**n= 3**	**n=1**	**n=4**	**n=6**	**n=2**	**n=1**	**n=4**
**RPE**							
Disposable face mask	0	0	0	0	0	0	1
Powered or air-fed. filtering respirator	0	0	0	0	0	0	1
Reusable half or full-face mask respirator (without powered or air-fed respirator)	1	1	3	2	2	0	0
Reusable half or full-face mask respirator	1	0	0	0	0	1	0
No RPE	0	0	0	2	0	0	0
No available data	1	0	1	2	0	0	2
**Gloves**							
Disposable gloves	1	0	2	4	0	0	1
Reusable gloves	1	0	1	1	1	1	1
Reusable and disposable gloves	0	1	0	0	1	0	0
No gloves	0	0	0	0	0	0	0
No available data	1	0	1	1	0	0	2
**Coveralls and other protective equipment**							
Protective glasses	0	0	0	0	0	0	1
Protective glasses, protection suit, safety footwear	1	0	1	1	1	1	0
No available data	2	1	3	5	1	0	3
**Ventilation - LEV**							
Yes	2	0	3	3	0	0	2
No	1	1	1	3	2	1	1
No available data	0	0	0	0	0	0	1

RPE = Respiratory protective equipment; LEV = Local exhaustion ventilation; n= number of workers performing the task.

Table S9. Detailed characteristics regarding the use of RPE, gloves, coveralls and other protective equipment, and presence of local exhaustion ventilation (LEV), for maintenance and laboratory work

Activity – Thermal spraying	Cleaning and maintenance of equipment	Machining operations (grinding) on parts containing chromium	Waste management	Other tasks
	**n=2**	**n=1**	**n=1**	**n=4**
**RPE**				
Disposable face mask	1	0	0	3
Powered or air-fed. filtering respirator	0	0	0	1
Reusable half or full-face mask respirator (without powered or air-fed respirator)	0	0	0	0
Reusable half or full-face mask respirator	1	1	1	0
No RPE	0	0	0	0
No available data	0	0	0	0
**Gloves**				
Disposable gloves	1	0	0	1
Reusable gloves	0	0	0	0
Reusable and disposable gloves	1	0	1	0
No gloves	0	0	0	1
No available data	0	1	0	2
**Coveralls and other protective equipment**				
Protective glasses	0	0	0	2
Coveralls	1	1	0	0
Protection suit	0	0	0	1
No available data	1	0	1	1
**Ventilation - LEV**				
Yes	1	1	1	0
No	1	0	0	0
No available data	0	0	0	4

RPE = Respiratory protective equipment; LEV = Local exhaustion ventilation; n= number of workers performing the task.

Table S10. Influence of variables "home location", "home-traffic density" and "smoking status" on total urinary chromium levels (pre-shift and post-shift).

**Null hypothesis:**	The distribution of post-shift levels of Total U-Cr is the same across categories
**Test:**	Independent samples Mann-Whitney test or Kruskal-Wallis test

**Dependent variables**		**Independent variables**		
		**Home location**	**Home - traffic density**	**Smoking status**
		**Sig**	**Sig**	**Sig**
**Welding**	**Pre-shift total U-Cr**	0.072	0.197	0.080
	**Post-shift total U-Cr**	0.510	0.836	0.836
**Bath plating**	**Pre-shift total U-Cr**	0.010	0.576	0.326
	**Post-shift total U-Cr**	0.026	0.771	0.938
**Painting**	**Pre-shift total U-Cr**	0.793	0.890	0.226
	**Post-shift total U-Cr**	0.493	0.786	0.323
**Machining**	**Pre-shift total U-Cr**	0.518	0.269	0.288
	**Post-shift total U-Cr**	0.465	0.849	0.235

Sig=Significance (level of significance was 0.05). Yellow cells = $p<0.05$.
